# Evolution of Energy Sources in Surgical Ablation of Atrial Fibrillation: Current Technology and Future Directions

**DOI:** 10.31083/RCM51123

**Published:** 2026-06-26

**Authors:** M. Cassandra Witt, James R. Edgerton, Matthew R. Schill, Ralph J. Damiano, Christian W. Zemlin

**Affiliations:** ^1^Division of Cardiothoracic Surgery, Washington University School of Medicine, Barnes-Jewish Hospital, St. Louis, MO 63110, USA

**Keywords:** maze procedure, atrial fibrillation, radiofrequency ablation, cryoablation, pulsed-field ablation

## Abstract

Atrial fibrillation (AF) is the most common sustained arrhythmia worldwide and is associated with substantial morbidity, increased stroke risk, and a significant healthcare burden. While antiarrhythmic medications and catheter ablation remain central to rhythm control strategies, the efficacy of these therapies declines in persistent and long-standing persistent AF. Surgical ablation has consistently demonstrated superior long-term rhythm control, but this approach remains underutilized due to the associated perceived invasiveness and procedural complexity. Meanwhile, advances in energy sources have transformed the field, with bipolar radiofrequency (RF) and cryoablation established as the gold standard for lesion creation, supported by decades of durable outcomes. Nonetheless, both modalities possess limitations related to thermal injury and technical demands. Pulsed-field ablation (PFA), a non-thermal modality that induces irreversible electroporation (IRE), represents a promising new direction. Indeed, PFA preferentially ablates cardiomyocytes while sparing adjacent structures, enabling rapid, reproducible lesion formation with an improved safety profile. Preclinical studies have demonstrated the ability of this technology to create transmural lesions. Meanwhile, catheter-based studies have shown the associated efficacy and safety with this technology, and early feasibility trials suggest that surgical PFA clamps can replicate most of the Cox-Maze lesion set. Current surgical ablation lesion sets rely primarily on RF or cryoablation. However, emerging modalities such as PFA are poised to enable effective epicardial ablation on the beating heart, a challenge for thermal-based ablation technologies. With the advent of hybrid strategies that combine epicardial surgical ablation with catheter-based endocardial ablation, PFA may broaden the role of surgical ablation in advanced AF.

## 1. Introduction

Atrial fibrillation (AF) is the most common sustained cardiac arrhythmia. Its estimated global prevalence was 50 million in 2020, with projections suggesting further increases over the coming decades [1]. AF frequently occurs in the setting of hypertension, diabetes, heart failure, and valvular disease, and its incidence increases with advancing age [1,2,3]. Although some patients are asymptomatic, AF can cause palpitations, fatigue, dyspnea on exertion, exercise intolerance, and diminished quality of life and is associated with a higher risk of stroke, heart failure, and mortality [1]. In addition, AF contributes to increased rates of hospitalization and long-term healthcare utilization, making it an important target for interventions aimed at improving both patient outcomes and healthcare costs [1].

Although antiarrhythmic medications are the foundation of rhythm-control therapy, their effectiveness diminishes in persistent and long-standing persistent AF, and adverse side effects limit long-term tolerance [1]. Catheter ablation has therefore emerged as an important therapeutic strategy, offering improved maintenance of sinus rhythm and enhanced quality of life compared to pharmacological therapy, particularly in paroxysmal AF [4,5,6]. Catheter ablation is now a Class I recommendation in patients with AF and concurrent heart failure with reduced ejection fraction, and in patients with symptomatic paroxysmal or persistent AF [1,7]. The effectiveness of catheter ablation declines in long-standing persistent AF, which is thought to be due to atrial remodeling and fibrosis [8,9]. Long-term follow-up studies in these patients have shown recurrence rates over 50%, even after multiple ablation procedures, highlighting the need for more effective and durable rhythm control interventions [10,11].

Surgical ablation has consistently shown higher long-term success rates than catheter ablation, particularly in patients with persistent and long-standing persistent AF [12,13,14,15]. The original “cut-and-sew” Cox-Maze procedure (CMP) established proof of concept by creating a comprehensive biatrial lesion set that, in most patients, restored sinus rhythm [16]. Its later modification, the CMP IV, replaced most of the incisions with bipolar radiofrequency (RF) or cryoablation, reducing operative complexity and recovery time while maintaining efficacy [12,17]. It is the only surgical procedure to receive a Food and Drug Administration (FDA) indication to treat AF during concomitant cardiac surgery [18]. Over the past two decades, refinements including mini-thoracotomy, thoracoscopic, and robotic-assisted approaches have limited the invasiveness of surgical rhythm-control strategies [19,20,21]. Emerging technologies may address residual limitations of thermal modalities. Pulsed-field ablation (PFA) is discussed in greater detail due to its rapid clinical development and expanding body of evidence. This review provides a comprehensive narrative review of surgical AF ablation devices with a focus on new and emerging technologies. It outlines the historical development of surgical lesion sets and reviews established and emerging energy sources, highlighting their strengths and limitations.

## 2. Historical Perspective: Cox-Maze to Modern Surgical Ablation

The development of surgical ablation for AF was driven by the concept that AF is sustained by multiple reentrant waves propagating throughout the atria [22]. Early operative strategies, such as left atrial isolation and the corridor procedure, attempted to interrupt conduction pathways by isolating portions of the atria [23]. While these approaches demonstrated proof of principle, they yielded incomplete efficacy and impaired atrial transport function, limiting their adoption.

**Fig. 1.**
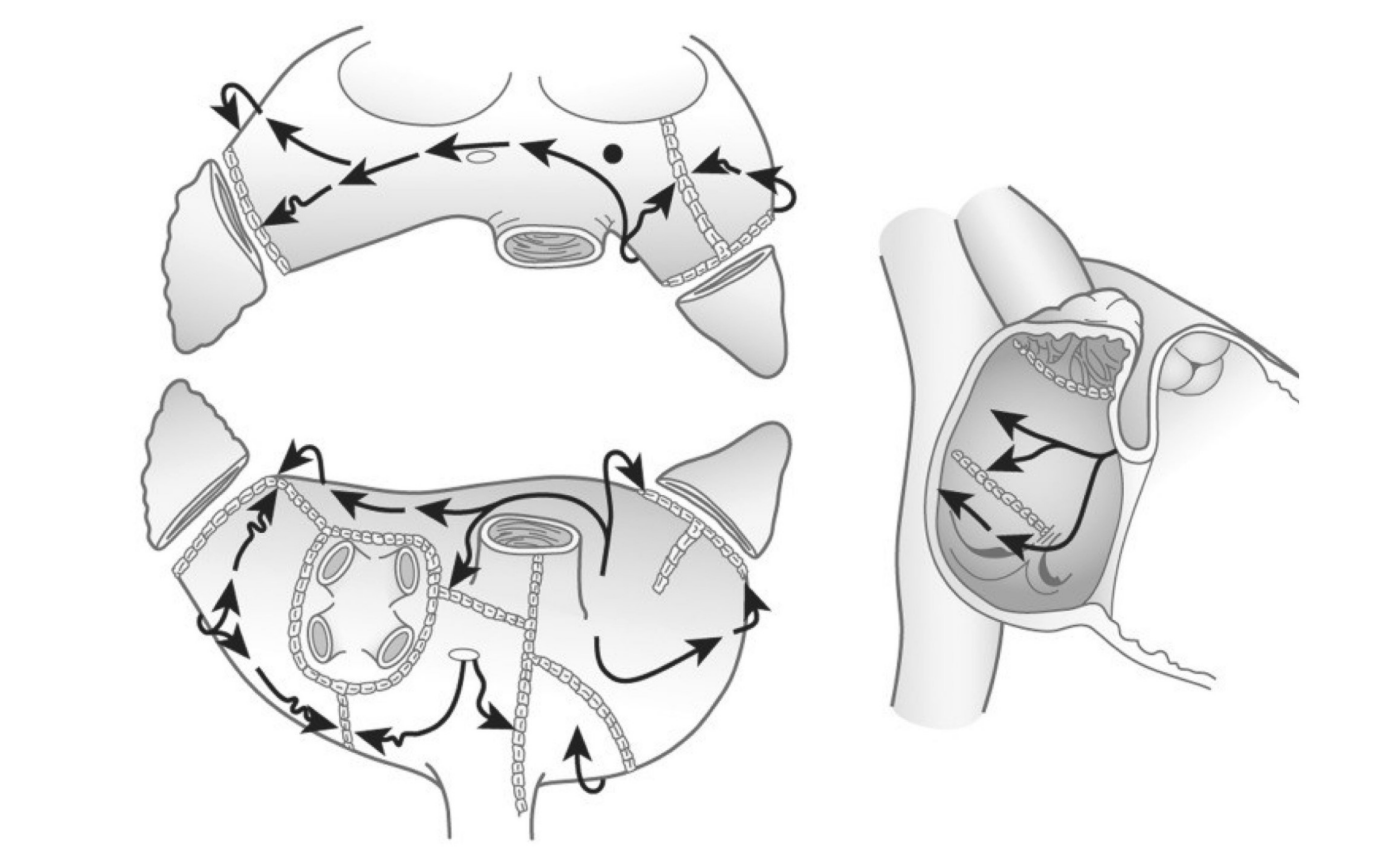
**The original ‘cut-and-sew’ Cox-Maze procedure**. Reproduced from Ref. [13] with permission from Circulation: Arrhythmia and Electrophysiology.

A major breakthrough occurred in 1987, when James Cox and colleagues introduced the CMP. By creating a series of carefully designed atrial incisions, the operation disrupted conduction pathways while preserving atrial contractility (Fig. 1, Ref. [13]). Clinical series demonstrated long-term freedom from AF rates exceeding 90%, establishing the “cut-and-sew” CMP as the first effective surgical treatment for AF [24,25,26,27]. Subsequent refinements led to the CMP III, which optimized the atrial lesion set, improving both efficacy and safety while preserving atrial function [24,28,29]. However, the complexity and technical demands of the procedure and prolonged cardiopulmonary bypass times limited its use to a small number of specialized centers.

**Fig. 2.**
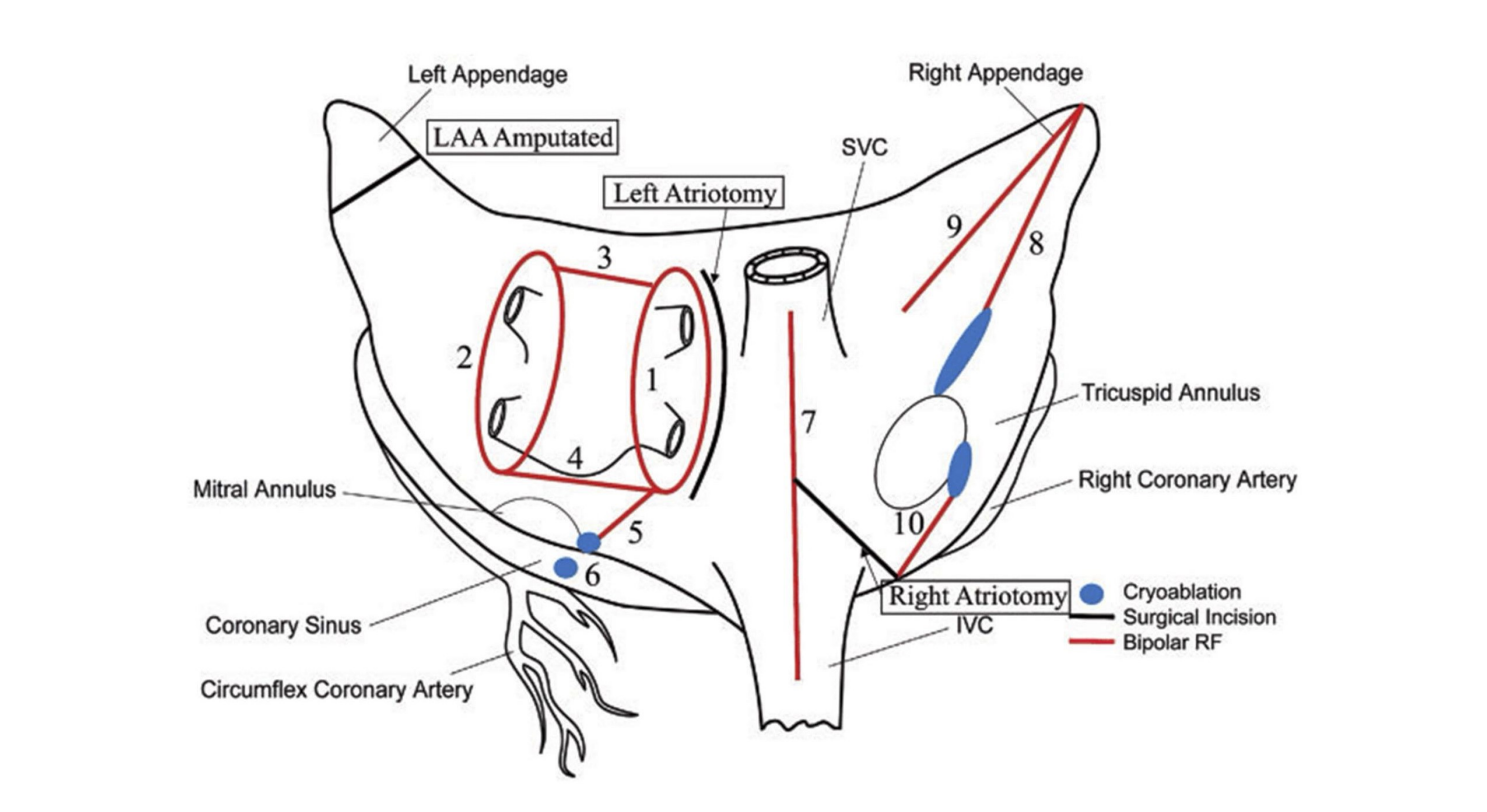
**Cox-Maze IV lesion set**. Left-sided lesions: (1) right pulmonary vein lesions; (2) left pulmonary vein lesions; (3) superior connecting lesion (roof); (4) inferior connecting lesion (floor); (5) mitral annulus lesion; (6) coronary sinus lesion. Right-sided lesions: (7) SVC to IVC lesions; (8) tricuspid valve lesions (10 o’clock); (9) right atrial free wall lesion; (10) tricuspid valve lesions (2 o’clock). IVC, inferior vena cava; LAA, left atrial appendage; RF, radiofrequency; SVC, superior vena cava.

The introduction of the CMP IV procedure in 2002 addressed many of these limitations by replacing atrial incisions with thermal ablation lines created using either bipolar RF or cryoablation (Fig. 2) [17]. These modalities were used since they proved to be the most effective energy sources tested in preclinical studies [30]. This transition simplified the operation, reduced cross-clamp and bypass times, and lowered perioperative morbidity while maintaining outcomes comparable to the CMP III [13,31]. As a result, the CMP IV became the most widely adopted iteration of the operation and remains a benchmark against which newer approaches are evaluated. It is the only surgical procedure, including all previous versions of the CMP, that has received FDA approval for the surgical treatment of AF [18].

Efforts to reduce invasiveness have also broadened surgical applicability. Minimally invasive strategies, such as right mini-thoracotomy and thoracoscopic techniques, enable lesion sets to be performed without sternotomy, resulting in reduced postoperative pain, shorter recovery, and improved cosmetic outcomes [20,32,33]. Robotic platforms have been utilized with success and have offered enhanced visualization for precise lesion delivery [19,34,35].

More recently, hybrid approaches have combined the strengths of surgical ablation with catheter ablation. These procedures typically involve epicardial surgical ablation followed by endocardial catheter-based mapping and touch-up ablation to ensure lesion transmurality and durability [36,37,38]. Early data from randomized trials suggest that hybrid ablation may improve outcomes in patients with persistent and long-standing persistent AF, compared to catheter ablation alone [10,36,37]. The evolution from the original cut-and-sew CMP to modern surgical and hybrid procedures reflects continuing efforts to maximize efficacy, safety, and accessibility in the treatment of AF.

## 3. Energy Sources in Surgical Ablation

The shift from cut-and-sew atriotomies to energy-based lesion creation represented an important development in the surgical management of AF. By creating transmural lesions without incisions, energy sources reduced procedural complexity and shortened operative times [39]. RF and cryoablation remain the most widely used and clinically validated energy sources. Other thermal methods, including microwave, laser, and high-intensity focused ultrasound, have been explored but abandoned due to safety and efficacy concerns [30,40,41].

### 3.1 Radiofrequency Ablation

RF has been one of the primary energy sources for surgical ablation. RF operates by delivering alternating current, typically in the range of 500–1000 kHz, to myocardial tissue. At these frequencies, the current causes localized heating but oscillates too rapidly to trigger myocyte depolarization. Temperatures above 50 °C cause irreversible coagulation necrosis. Resistive heating occurs throughout the tissue in proportion to the current density. Because the current is most concentrated at the zone of direct contact, lesion depth depends on conductive heat transfer into the surrounding myocardium [40]. RF ablation is impeded by factors such as air, epicardial fat, and tissue char formation, all of which can disrupt energy delivery and limit lesion depth.

In surgical practice, RF can be applied with unipolar or bipolar systems. It can also be dry or irrigated. Unipolar RF delivers current from a single electrode through the atrial tissue to a grounding pad, resulting in a wide energy pathway [42]. Unipolar RF devices are available in multiple configurations, including pens, linear flexible electrodes, and suction-assisted probes, with options for irrigation to enhance lesion depth and limit char formation. Unipolar RF has struggled to achieve consistent transmurality, particularly when applied epicardially. The circulating blood provides a convective heat sink that has prevented the sub-endocardium from reaching lethal temperatures and has led to gaps that can sustain conduction [40,43]. Cardiopulmonary bypass can mitigate this limitation by decreasing convective cooling from the circulating intracavitary blood, but even then, lesion depth has been unpredictable and requires meticulous overlapping applications to approximate linear lesions. For these reasons, unipolar RF has largely been replaced by bipolar RF for surgical ablation. However, unipolar RF has found a role in hybrid approaches, as the lack of transmurality can be corrected from an endocardial approach.

**Fig. 3.**
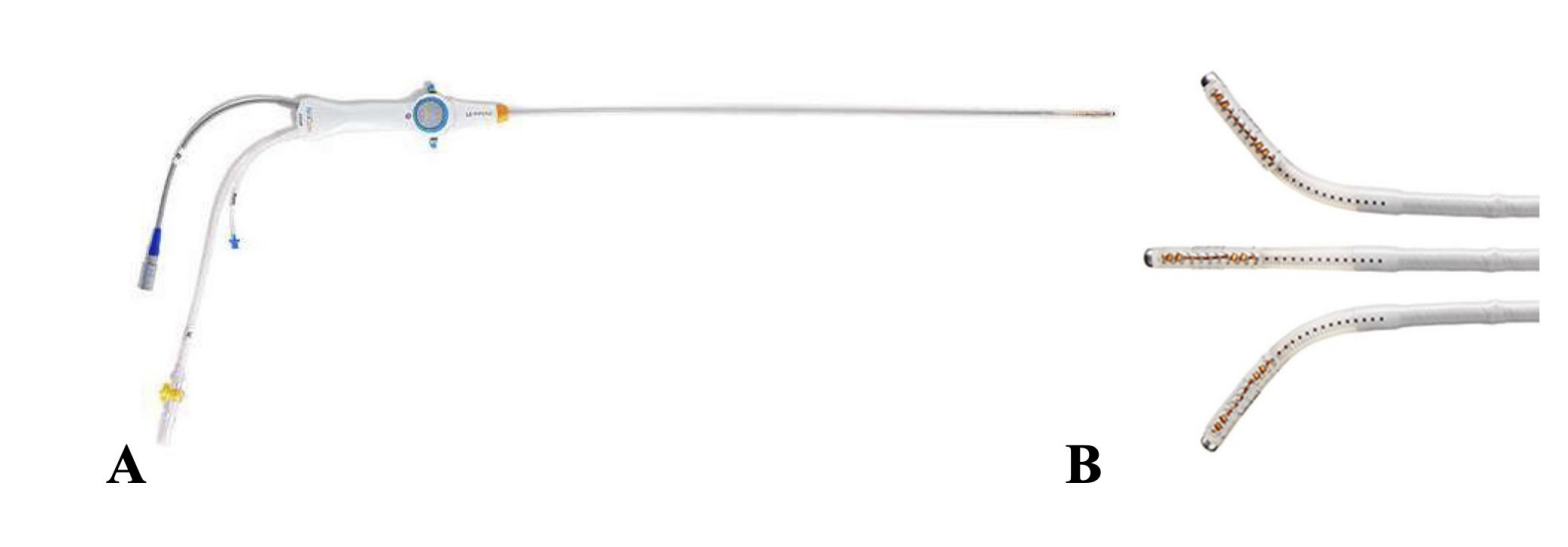
**The Epi-Sense^TM ^ epicardial ablation device**. (A) Overview of the Epi-Sense^TM^ device and delivery system. (B) Magnified image showing the helical RF coil and integrated suction channels that stabilize the probe during lesion creation. Reproduced with permission from AtriCure.

The only unipolar RF device currently used in clinical surgical ablation is the EPi-Sense^TM^ (AtriCure, Cincinnati, OH, USA) ablation catheter, which serves as the epicardial component of the CONVERGENT procedure (Fig. 3). This suction-assisted, irrigated electrode has been designed to improve tissue contact during epicardial ablation along the posterior left atrium (LA) [36]. Although it has received FDA approval as part of a hybrid procedure, it has not been used as a standalone epicardial device, due to limited efficacy at creating transmural lesions [30].

Bipolar RF addressed many of these limitations by confining the current between two electrodes applied on either one or both sides of the atrial tissue [42]. Bipolar devices can be either directional or constrained. Directional bipolar devices contain side-by-side electrodes positioned on the epicardial tissue surface. Constrained bipolar devices use a clamp configuration with opposing jaws that deliver energy from opposite sides of the atrial wall [2]. They ensure uniform contact while isolating the myocardium from the circulating blood, allowing focused energy delivery, producing lesions that are reproducible and reliably transmural [44,45,46].

Bipolar RF clamps incorporate impedance-based feedback algorithms that adjust power in real time and stop delivery when resistance reaches a plateau. Studies in explanted human hearts have shown that double ablations without unclamping markedly improved the likelihood of continuous transmurality, underscoring the importance of technique in addition to device design [44,46]. Practical considerations to ensure adequate delivery of RF to the tissue, including electrode cleaning to limit char formation, avoiding tissue folding or air pockets, which create a high resistance to current flow, and ensuring adequate contact force, remain critical for success.

The Coolrail^TM^ linear pen (AtriCure, Cincinnati, OH, USA) was developed as a directional bipolar RF device intended to create continuous, transmural epicardial lesions on the beating heart. Pre-clinical evaluations have demonstrated inconsistent lesion transmurality, particularly in areas of thicker atrial tissue [30,47]. These limitations have prevented the Coolrail^TM^ from widespread adoption for surgical AF ablation.

**Fig. 4.**
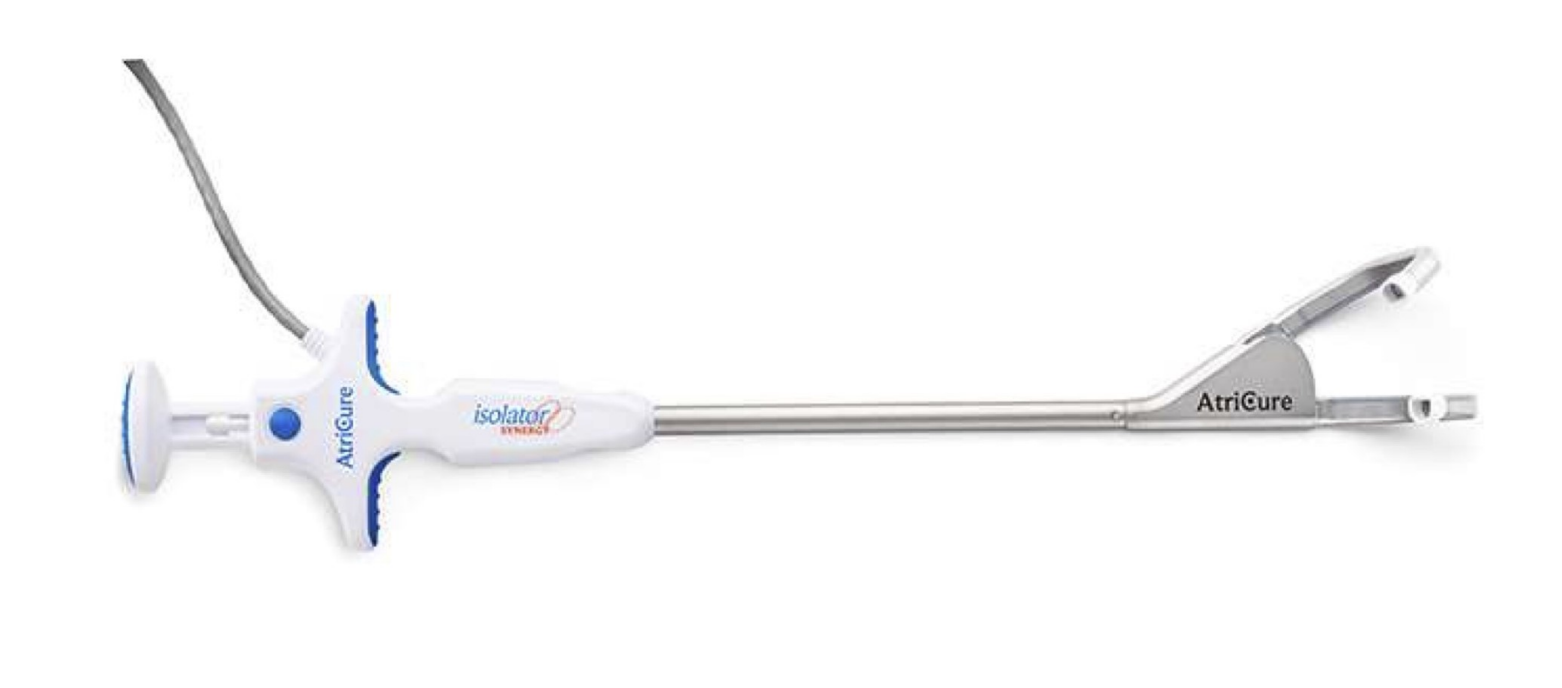
**EnCompass^TM^ bipolar RF ablation clamp**. Reproduced with permission from AtriCure.

Another innovation has been the EnCompass^TM^ (AtriCure, Cincinnati, OH, USA) clamp, a constrained device engineered to create the “box” lesion around the pulmonary veins and posterior LA in a single application (Fig. 4). While early experimental data have been promising [45], clinical validation remains ongoing [48]. This design reflects the recognition that durable posterior wall isolation is central to long-term rhythm control. In a clinical study of the Cox-Maze lesion set without a complete box isolation of the entire posterior LA and all 4 pulmonary veins, the rest of the CMP had only a 33% success rate at 5 years [12].

In practice, RF ablation has been integrated into the CMP IV and minimally invasive or hybrid strategies. During on-pump operations, bipolar clamps are used to isolate pulmonary veins, create left atrial roof and floor lines, and complete right atrial lesions in a matter of 10–40 seconds per application [49,50]. Off-pump use is possible but requires caution, and historically has been confined to pulmonary vein isolation (PVI) alone, which did not have durable long-term success in preventing AF. Recently, bipolar clamps have been used to fully isolate the entire posterior LA with much improved results [10,37]. Additional lesions, attempted with unipolar and directional bipolar RF, have had limited success in a surgical setting, but have shown efficacy in hybrid procedures such as the CONVERGENT procedure [36].

A disadvantage of unipolar RF has been the incidence of collateral thermal damage to surrounding vital structures, such as coronaries, valve tissue, the phrenic nerve, and the esophagus [51]. These are virtually never seen with constrained bipolar RF devices, which is a major advantage of this technology.

The wide variety of available devices makes RF ablation highly adaptable across a spectrum of surgical situations. Regardless of the specific device, the principles of bipolar RF ablation remain central to its effectiveness and explain why it has become a cornerstone of many surgical ablation procedures.

### 3.2 Cryoablation

Introduced in cardiac surgery in 1951, cryoablation was first explored as a treatment of ventricular tachycardia before becoming a key component of surgical AF ablation [52]. It has proven valuable in many procedures [17,53,54]. Cryoablation can be used as the sole energy source or complementary to RF, particularly in anatomic regions where collateral injury could result in injury to vital tissue. Unlike RF, which generates lesions through resistive heating, cryoablation relies on controlled tissue cooling to achieve irreversible cellular injury. When cardiac tissue is cooled to approximately –30 °C to –40 °C, intracellular and extracellular ice crystals form, disrupting the cell membrane and organelles [55]. During the freeze-thaw cycle, osmotic stress causes fluid shifts, ice recrystallization, and membrane rupture, leading to cell death while preserving the extracellular matrix and overall tissue architecture [42,56,57]. Unlike heat injury, which denatures structural proteins such as collagen, freezing preserves these proteins, leaving the extracellular matrix intact. These characteristics make cryoablation especially valuable in areas where tissue integrity must be maintained, such as the valve annuli and leaflets.

The freeze-thaw cycle is central to cryoablation’s effectiveness. As the cryoprobe removes heat, ice forms first outside the cells, drawing water out and causing dehydration, and inside the cells, rupturing membranes and producing immediate cell death near the probe. During thawing, recrystallization and osmotic swelling add further injury, while reperfusion triggers delayed necrosis and apoptosis at the lesion margins [55,58]. The most effective lesions result from a rapid freeze, followed by a slow thaw, which together maximize both the immediate and delayed damage while preserving tissue integrity [57].

**Fig. 5.**
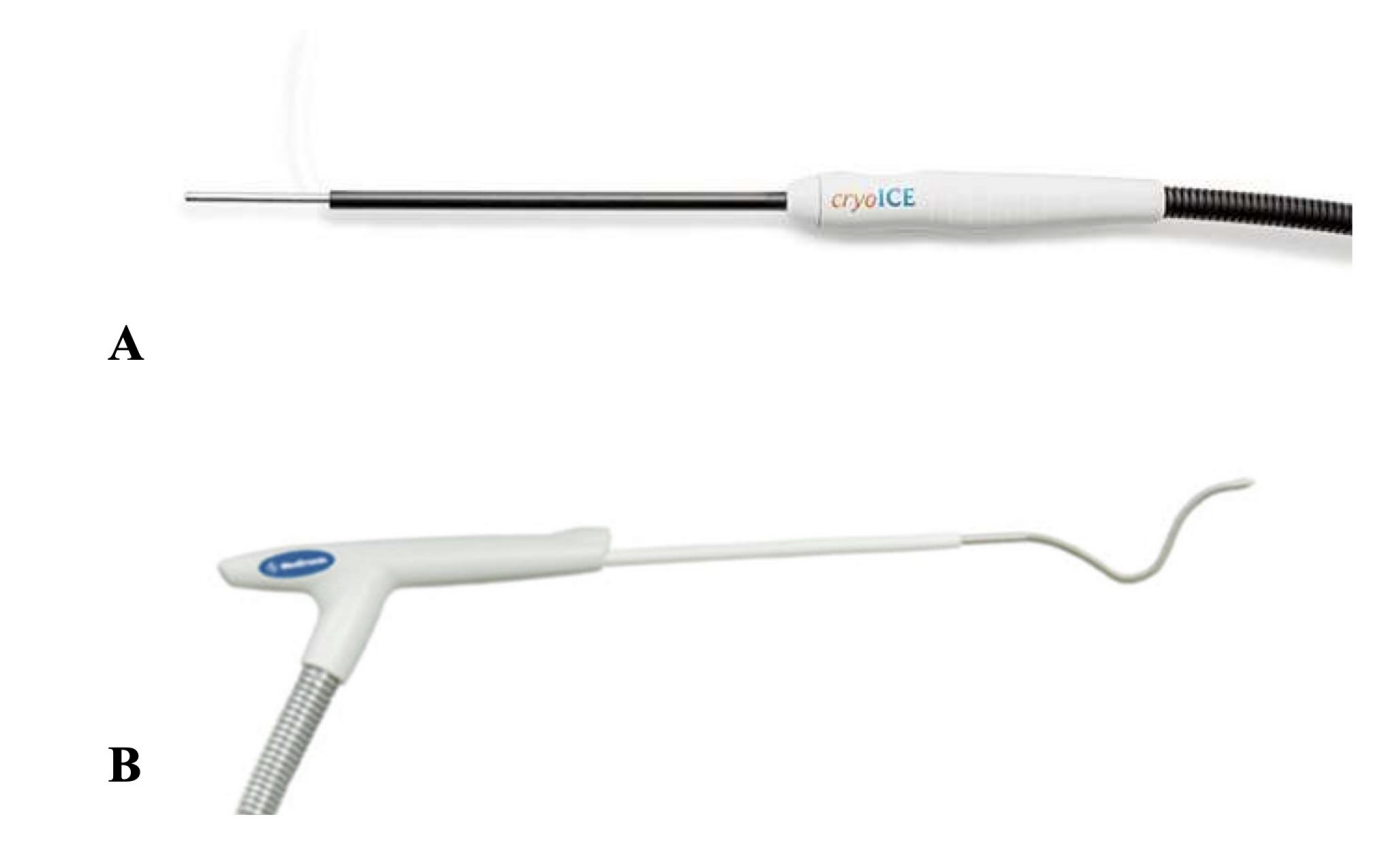
**Surgical cryoablation probes**. (A) CryoICE cryoablation probe. (B) CryoFlex^TM ^flexible cryoablation probe. Reproduced with permission from AtriCure and Medtronic.

Currently, two manufacturers produce cryoablation devices for surgical cardiac ablation. The cryoICE system (AtriCure, Cincinnati, OH, USA) uses nitrous oxide to cool tissue to approximately –50 °C to –70 °C, while the Cardioblate, CryoFlex^TM^, and CryoFlex^TM^ 10-S clamp systems (Medtronic, Minneapolis, MN, USA) use argon to reach temperatures as low as –160 °C (Fig. 5). Both systems employ disposable, malleable cryoprobes that can be shaped to facilitate precise, conformal contact with atrial tissue [59]. Freezing times are between 2 and 3 minutes.

Although cryoablation produces highly reproducible lesions, several limitations remain. The relatively lengthy freezing times increase procedural duration. Lesion depth is influenced by factors such as tissue thickness, the presence of epicardial fat, and local perfusion [57,58]. Because circulating intracavitary blood acts as a strong heat sink, effective epicardial cryoablation has not been possible, making cardiopulmonary bypass necessary and limiting its use in minimally invasive approaches. In the clinical setting, epicardial fat prevents adequate energy penetration, requiring endocardial probe placement. In addition, successful cryoablation depends on tissue contact with the probe. Even small gaps can result in ice ball formation between the probe and the tissue, which can lead to non-transmural lesions.

Collateral damage to surrounding structures is also possible and should be carefully avoided. While proven safe on valve tissue, it has been implicated in causing late coronary arterial injury and intimal hyperplasia. It should not be used over the coronary arteries. It can also result in phrenic nerve injury [59,60]. Despite these challenges, cryoablation has secured its place as a cornerstone of surgical AF ablation, offering a reliable balance between lesion durability and preservation of structural integrity.

### 3.3 Pulsed-Field Ablation

PFA differs from RF and cryoablation in that it is designed to induce cell death via irreversible electroporation (IRE), a mechanism that does not require changes in temperature. Cell death results from the creation of permanent membrane pores that disrupt cellular homeostasis. PFA applies high-voltage electrical pulses delivered over a very short duration, nanoseconds to microseconds, which destabilize the lipid bilayer of the cell membrane, creating nanopores. IRE results in loss of membrane integrity, ion homeostasis disruption, and loss of osmotic balance, which ultimately leads to cell death through necrosis and apoptosis. While some cellular injury occurs immediately following energy delivery, lesion maturation may also involve delayed apoptotic and necrotic mechanisms, with tissue remodeling evolving over days to weeks [61,62]. Pore formation is the primary mechanism of cell death, but other factors also contribute. These include lipid peroxidation, changes in pH, and the generation of reactive oxygen species [63,64]. When appropriately delivered, PFA preferentially injures cardiomyocytes while preserving the extracellular matrix, thus maintaining tissue architecture.

First attempts at IRE with direct current (DC) ablation in the 1980s and 1990s were limited by involuntary skeletal muscle contractions and significant patient discomfort [65]. Modern PFA technologies differ from the early systems through the use of precisely timed, high-frequency biphasic waves that deliver energy in a more controlled manner. This advancement has largely eliminated the muscle contractions and pain associated with early IRE. In 2019, Reddy et al. [66] found that among 81 patients treated with catheter-based PFA under general anesthesia, none experienced skeletal muscle contractions. In a separate cohort of 106 patients undergoing biphasic PFA under conscious sedation, only mild, well-tolerated muscle activation was observed [67].

The effectiveness of PFA depends on the parameters used to deliver IRE, including waveform morphology, voltage, pulse duration, interval between pulses, and the number of pulses delivered. These variables shape lesion size, depth, and uniformity, highlighting the need for optimized protocols to maximize efficiency and minimize collateral injury [68,69,70]. Unlike thermal ablation, PFA is to some degree tissue selective. This advantage provides a safety margin not possible with RF or cryoablation. Cardiomyocytes have a lower electroporation threshold than surrounding tissues, allowing precise ablation of atrial myocardium while theoretically sparing adjacent structures [71,72,73]. With properly chosen parameters, lethal specificity for myocardial cells can be achieved, and collateral damage to the esophagus, phrenic nerves, coronary arteries, and pulmonary veins can be avoided. Experimental data suggest the presence of mixed zones of irreversible and reversible electroporation that cannot be reliably distinguished in the acute setting and may have implications for lesion durability [71].

#### Safety Profile and Efficiency of PFA

PFA has demonstrated a strong safety profile. Preclinical and clinical catheter-based studies have shown that PFA minimizes collateral injury to surrounding structures. Pulmonary vein stenosis, a known complication of unipolar RF ablation caused by heat-induced vascular remodeling, has not been observed following PFA in either animal or early human studies [65,74,75].

One problem has been the induction of coronary spasm with resultant myocardial ischemia. Clinical studies have similarly demonstrated that PFA delivered near coronary arteries can provoke marked but reversible vasospasm, whereas PFA applied at sites remote from the coronary circulation has not elicited this response [76]. In a chronic porcine model of surgical PFA in our laboratory, two of nine animals developed sudden ventricular fibrillation within one second of energy delivery across the atrioventricular groove and the coronary arteries. These arrhythmias were refractory to multiple defibrillation attempts and were attributed to coronary vasospasm since histologic assessment showed no significant injury to coronary arteries or valves [77].

Phrenic nerve injury, typically seen with ablation near the right pulmonary veins, has been absent or transient in animal studies even with intentional targeting [78]. Esophageal injury has also been minimal, with limited and transient injury reported and no evidence of lasting injury in chronic animal studies [79,80]. In addition, PFA lesion formation depends on electric field strength rather than tissue heating, eliminating risks of injury associated with charring and steam pops [81]. Concerns have been raised about bubble formation and arcing at the electrode-blood interface. However, studies using energy up to 200 J have reported no significant arcing and only minimal, transient microbubble formation [65,66,82].

Acute kidney injury has also been reported in catheter-based PFA and is believed to be mediated by procedure-related hemolysis. Experimental and clinical studies have demonstrated that biochemical hemolysis occurs in a dose-dependent manner and can be detected even after a limited number of applications, with severity increasing with lesion number and reduced catheter tissue contact [83]. In mechanistic models, free plasma hemoglobin rose linearly with repeated PFA deliveries and was observed after as few as two applications, confirming hemolysis as an intrinsic effect of PFA energy delivery [84]. Large comparative clinical series further demonstrated that hemolysis is common and varies across catheter platforms, highlighting important technology-dependent effects [84]. In a prospective series of 115 patients, biochemical hemolysis was common, while acute kidney injury (AKI) occurred in only 7% of patients, with just one case resulting in meaningful renal dysfunction. The number of PFA applications did not differ between patients with and without AKI, and modeling suggested 129–140 pulses were required to produce a creatinine rise meeting AKI criteria [85]. Larger registry data similarly showed that clinically significant renal injury was rare, occurring in fewer than 0.05% of patients [86]. Together, these findings underscore hemolysis and renal dysfunction as pulse- and device-dependent phenomena and an evolving safety consideration as PFA technologies advance.

Beyond safety advantages, PFA offers procedural efficiency. Lesion formation is nearly instantaneous, as electroporation disrupts cell membranes within nanoseconds. In the initial clinical series of catheter-based PFA, average procedure duration was 67 minutes, substantially shorter than the 96 to 166 minutes typically reported for thermal techniques [75,87].

Because PFA is a relatively new approach to AF ablation, its long-term outcomes and limitations remain incompletely defined. It remains uncertain whether IRE lesions include areas of reversible injury similar to those seen with RF. With thermal energy, these partially injured zones may manifest temporary conduction block that later recovers, contributing to AF recurrence [88]. Whether PFA produces a comparable pattern of transient uncoupling or achieves consistently irreversible injury is not yet clear, underscoring the need for further investigation in both preclinical and clinical studies.

## 4. Clinical Outcomes With Thermal Ablation

Clinical outcomes in AF ablation depend on multiple factors, including the energy modality, the lesion set, and the underlying atrial substrate. Although most randomized controlled trials have evaluated catheter ablation, surgical ablation series and registry reports have provided robust evidence that surgical ablation achieves higher freedom from AF, especially in patients with persistent and long-standing persistent AF [12,15]. The CMP is considered the gold standard lesion set [31].

### 4.1 Stand-Alone Surgical Ablation

Stand-alone surgical ablation, particularly the CMP IV, has demonstrated excellent long-term efficacy and safety for patients with symptomatic, refractory AF, including persistent and long-standing persistent AF. Multiple series have reported freedom from AF of approximately 92%, 84%, and 77% at 1, 5, and 10 years, with most patients maintaining sinus rhythm off antiarrhythmic drugs and anticoagulation [14,21,89,90]. Across multiple series totaling 543 patients undergoing stand-alone Cox Maze III or IV procedures for refractory AF, there were no reported operative mortalities. Major complications included cerebrovascular accident (0.8%–1.7%), renal failure requiring dialysis (0%–0.8%), pneumonia (2.5%–2.8%), reoperation for bleeding (0.4%–1.5%), and permanent pacemaker implantation (4%–8%) [14,21,90]. Minimally invasive approaches have yielded similar outcomes to sternotomy, with a reduction in morbidity [21,90]. Predictors of late recurrence have included longer preoperative AF duration, increased left atrial size, and multiple prior catheter ablation failures [14,21,90]. Our institutional experience has been consistent with these findings. In our most recent series (n = 236), freedom from recurrent atrial tachyarrhythmias (ATAs) was 94% at 1 year and almost 80% at 10 years, with similar efficacy observed in patients with paroxysmal and non-paroxysmal AF. Perioperative mortality has remained low, with no operative deaths over two decades [14,90].

Guidelines from the 2024 Heart Rhythm Society (HRS) consensus statement support stand-alone surgical ablation for symptomatic patients with persistent AF with prior unsuccessful catheter ablation who are refractory to medical therapy [2]. Quality of life measures have been improved following the procedure, and most patients have been able to stop anticoagulation when durable sinus rhythm has been achieved [14,89]. Overall, stand-alone CMP has offered durable rhythm control with an acceptable morbidity for appropriately selected patients with advanced or refractory AF.

### 4.2 Concomitant Surgical Ablation

Concomitant surgical ablation refers to the addition of surgical ablation for AF at the time of another cardiac surgical procedure. It has been shown to improve long-term rhythm control and reduce thromboembolic events. The 2023 Society of Thoracic Surgeons (STS) guideline gave a Class I, Level A recommendation for surgical AF ablation during first-time, nonemergent cardiac surgery, along with a Class I, Level A recommendation for left atrial appendage occlusion at the same operation. The 2023 American College of Cardiology/Heart Rhythm Society/American Heart Association (ACC/HRS/AHA) guideline offers a Class IIa recommendation that concomitant surgical ablation may be beneficial in patients with AF undergoing cardiac surgery to reduce the risk of recurrent AF, and a Class I, Level A recommendation for left atrial appendage exclusion in patients with elevated stroke risk when combined with anticoagulation [1].

Despite strong guideline support, real-world adoption of concomitant AF treatment at the time of cardiac surgery has remained limited. According to the 2023 STS guidelines, in 2022, only 43% of patients with documented AF undergoing first-time, nonemergent cardiac surgery received both surgical ablation and left atrial appendage occlusion, while 30% received neither therapy. The undertreatment of AF was most pronounced among patients undergoing isolated coronary artery bypass grafting or aortic valve replacement, whereas higher rates of both ablation and appendage management were observed in those undergoing mitral valve procedures [3].

Randomized trials and meta-analyses have demonstrated higher rates of sinus rhythm maintenance at one year when surgical ablation is added to mitral valve surgery. Freedom from AF, at one year, has ranged from 60% to 90%, depending on the lesion set employed and the underlying atrial substrate [1,3,91,92]. Similar benefits have been observed across mitral, aortic, and coronary artery bypass procedures [3]. Several studies have further demonstrated a reduction in long-term stroke risk when surgical ablation is performed concomitantly with cardiac surgery [93,94]. Patients with AF who undergo concomitant surgical ablation during cardiac surgery also have demonstrated markedly improved long-term survival, a 53% reduction in mortality compared to similar patients whose AF was left untreated [95]. However, randomized data have not shown a consistent reduction in all-cause mortality or heart failure hospitalization, although selected observational studies suggest potential survival benefits, particularly among patients who achieve a durable sinus rhythm [3,92,93].

Permanent pacemaker implantation has been observed following concomitant surgical ablation, with reported relative risks ranging from 1.3 to 2.7. This risk has been somewhat higher with biatrial lesion sets compared with left-sided ablation alone [1,3,91,92]. Some studies also reported an increased incidence of postoperative renal dysfunction, particularly in patients undergoing more extensive ablation or those with preexisting renal impairment [1]. Despite these risks, overall major complication rates, including bleeding, infection, and mortality, have been comparable to cardiac surgery performed without ablation [15,91,96]. Finally, quality-of-life outcomes and AF burden scores improve in patients who maintain sinus rhythm following concomitant surgical ablation, further supporting its clinical benefit [3,96].

### 4.3 Minimally Invasive Ablation

Efforts to reduce the invasiveness of surgical ablation have led to the development of mini-thoracotomy, thoracoscopic, and robotic-assisted ablation procedures, which aim to preserve the durability of traditional lesion sets while minimizing surgical trauma. Our group and others have demonstrated that the CMP IV can be performed using minimally invasive approaches with similar or better efficacy and lower morbidity [19,20,21].

Minimally invasive CMP IV performed via right mini-thoracotomy has achieved freedom from atrial tachyarrhythmias off antiarrhythmic drugs in 74%–81% of patients at 1–2 years, with complication rates and mortality lower than sternotomy approaches [20]. Five-year data show 73% of patients with persistent/long-standing persistent AF remain in sinus rhythm off antiarrhythmic drugs after a single intervention, with very low perioperative risk [21].

Robotic-assisted biatrial CMP has reliably replicated the classic lesion set [34]. In a series of 135 patients with persistent AF, robotic cryothermic CMP resulted in 97% freedom from arrhythmia at 9–12 months, with 2.2% operative mortality and no deaths in stand-alone cases. Stroke, repeat ablation, and need for anticoagulation were rare [19]. When combined with mitral valve surgery, robotic-assisted AF ablation has demonstrated low recurrence rates and excellent safety, with a 96% five-year survival [97].

Totally thoracoscopic PVI procedures have demonstrated lower AF-free success rates in randomized studies, ranging from 33% to 66% at one year [98,99]. Late outcomes generally have been even worse, particularly in non-paroxysmal AF [100]. This has led to recent enthusiasm for hybrid approaches.

### 4.4 Hybrid Ablation

Hybrid ablation combines minimally invasive surgical epicardial ablation with endocardial catheter ablation. The surgical phase involves thoracoscopic epicardial ablation, usually of the posterior left atrial wall and pulmonary veins, while endocardial catheter access is used to confirm lesion completeness and perform targeted touch-up of residual conduction gaps. Patient selection has focused on those with persistent or long-standing persistent AF, particularly patients who have failed prior catheter ablation. Hybrid procedures can be performed either simultaneously or in a staged fashion [101].

The CONVERGE trial (August 2016–December 2019) randomized 153 patients with persistent and long-standing persistent AF in a two-to-one fashion to the Convergent hybrid procedure or endocardial catheter ablation alone. Epicardial ablation was performed via a subxiphoid pericardioscopic approach using a unipolar RF catheter to create posterior wall ablation, followed by endocardial catheter ablation for PVI, mitral line completion, and confirmation of lesion continuity. At 12 months, freedom from AF in the hybrid group was 68% compared with 50% in the catheter group. Off antiarrhythmic drugs (AADs), freedom was 54% vs. 32%. Major adverse events occurred in 7.8%, including stroke (1), transient ischemic attack (TIA) (1), excessive bleeding (1), pericardial effusion (4), and phrenic nerve injury (1), of the hybrid group vs. 0% in the catheter group. The hybrid approach demonstrated superior effectiveness but was associated with a higher early procedural complication rate [36].

The HARTCAP-AF trial (January 2017–September 2018) randomized patients with persistent AF to a thoracoscopic hybrid surgical RF ablation procedure versus catheter ablation. The surgical component included thoracoscopic bipolar RF ablation of the pulmonary veins and a posterior wall lesion set, plus left atrial appendage exclusion. The endocardial phase addressed gaps and performed complete mapping and touch-up ablation. At 12 months, freedom from AF off AADs in the hybrid arm was 89% compared to 41% in the catheter-only group. The complication rate was not significantly different between groups (21% vs. 14%, *p* = 0.68) [37]. At three years, hybrid ablation maintained superior rhythm outcomes compared with catheter ablation alone, with freedom from atrial tachyarrhythmias of 57% vs. 23% off antiarrhythmic drugs and fewer repeat catheter procedures (3 vs. 15) [102].

The CEASE-AF trial (December 2015–November 2019) compared a thoracoscopic hybrid ablation strategy, including PVI, posterior wall isolation, and left atrial appendage closure, to endocardial catheter ablation in 154 patients. At 12 months, freedom from AF without AADs was 72% in the hybrid group versus only 39% in the catheter group. Major complication rates were 8% for the hybrid group and 6% for the catheter group, indicating no significant difference in safety [38].

Together, these industry-sponsored randomized trials have demonstrated that hybrid ablation provides superior rhythm control compared with catheter ablation alone in patients with persistent and long-standing persistent AF (Table 1, Ref. [36,37,38]). The hybrid approach has overcome some of the limitations of catheter ablation. Overall, hybrid ablation offers improved efficacy compared with catheter ablation alone while maintaining lower invasiveness than open surgical approaches for carefully selected patients. However, the studies were not powered to detect differences in major adverse complications, highlighting the importance of ongoing surveillance through larger prospective registries to better characterize long-term safety. It should be noted that these randomized hybrid ablation trials compare surgical epicardial ablation with thermal catheter ablation, and the relative efficacy and safety of hybrid strategies versus catheter-based PFA remains to be determined.

**Table 1.** **Comparison of CONVERGE, HART-CAP, and CEASE-AF hybrid ablation trials, detailing AF classification, lesion sets, surgical approaches, outcomes, and major complication rates**.

Trial	AF classification	Lesion set	Surgical approach	Catheter component	Primary outcomes at 12 months	Major complication rates
CONVERGE Trial [36]	Persistent and long-standing persistent AF	Posterior left atrial wall ablation plus PVI and mitral line	Subxiphoid unipolar RF posterior wall ablation	Endocardial PVI, mitral line, mapping, and gap closure	Freedom from AF 68% vs. 50% catheter ablation, Freedom off AADs 54% vs. 32%	8% hybrid vs. 0% catheter
HARTCAP AF Trial [37]	Persistent AF	Thoracoscopic PVI and posterior wall box plus LAA exclusion	Thoracoscopic bipolar RF	Endocardial mapping and touch-up ablation	Freedom from AF off AADs 89% vs. 41% with catheter ablation	21% hybrid vs. 14% catheter
CEASE AF Trial [38]	Persistent and long-standing persistent AF	Thoracoscopic PVI, posterior wall box, LAA closure, and linear lesions	Thoracoscopic bipolar RF	Endocardial PVI confirmation and gap ablation	Freedom from AF off AADs 72% vs. 39% with catheter ablation	7.8% hybrid vs. 5.8% catheter

AF, atrial fibrillation; PVI, pulmonary vein isolation; AADs, antiarrhythmic drugs.

## 5. Pulsed-Field Ablation: Preclinical and Catheter Ablation Outcomes

**Fig. 6.**
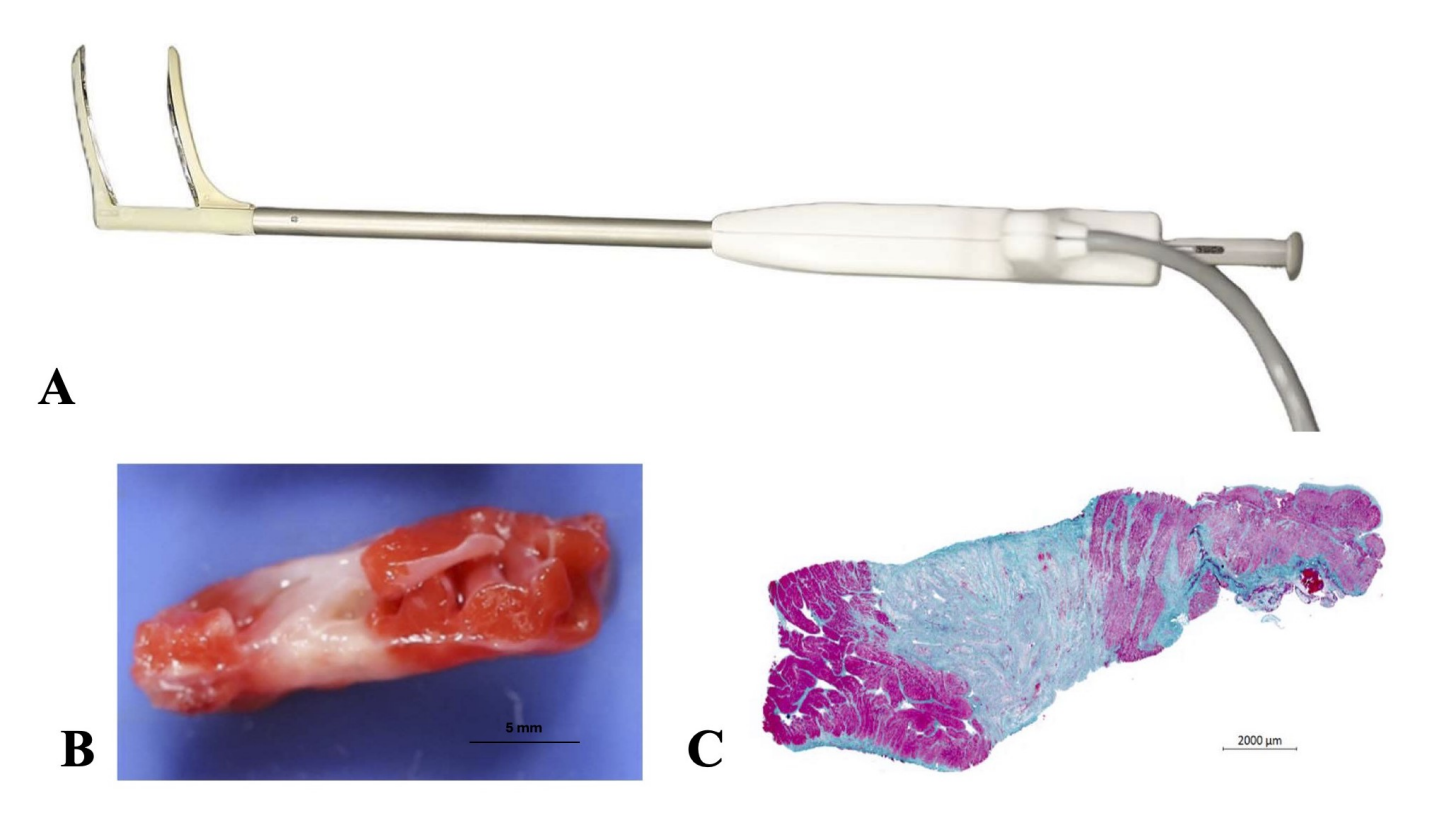
**Bipolar radiofrequency clamp and representative lesion formation**. (A) Bipolar radiofrequency clamp device. (B) Gross pathology specimen demonstrating lesion formation with a 5 mm scale bar. (C) Histologic section demonstrating transmural lesion formation with a 2000 μm scale bar.

Preclinical studies have provided the foundational evidence for PFA and its potential application in surgical AF ablation. Early porcine experiments demonstrated that epicardial clamps delivering biphasic electrical pulses could create transmural lesions across the atrial wall within seconds (Fig. 6) [103,104]. In an acute porcine model, using a parallel PFA clamp, nine lesions per animal were created in locations representative of the Cox-Maze lesion set, with a mean ablation time of only 2.5 seconds per lesion. Among 53 lesions examined histologically, transmurality was confirmed in 99%, and exit block was achieved in 96% of targeted sites, without any arrhythmias or procedural complications. Histologic analysis confirmed sharply defined necrosis confined to the myocardium [77,105].

In a chronic porcine model evaluating a novel nanosecond PFA clamp, animals underwent pulmonary vein and posterior left atrial ablation. An average of 35 days later, lesions were evaluated and demonstrated complete continuity and full transmurality. Durable conduction block was maintained across all targeted sites, and histologic analysis confirmed mature fibrotic scar formation [106]. In a subsequent porcine study, nine animals underwent biatrial nanosecond PFA. Seven animals survived to 26 days, while two died intraoperatively due to refractory ventricular fibrillation attributed to coronary artery vasospasm during ablation across the atrioventricular groove. Among surviving animals, histologic assessment demonstrated transmurality in 99% of lesion cross sections and 97% of individual lesions. Exit block was confirmed in 94% of the tested sites. No structural injury to coronary arteries or cardiac valves was identified [77].

Clinical experience with catheter-based PFA has rapidly expanded and validated the preclinical findings. Several randomized trials have evaluated catheter-based PFA for PVI. The ADVENT trial, a multicenter randomized study of more than 600 patients with paroxysmal AF, demonstrated non-inferiority of PFA compared with thermal ablation, with 12-month freedom from atrial arrhythmia of 73% vs. 71%, respectively [107]. Similarly, the SINGLE SHOT CHAMPION trial randomized 210 patients with paroxysmal AF to PFA or cryoballoon ablation and found a lower incidence of atrial tachyarrhythmia recurrence with PFA, 37% vs. 51%, confirming non-inferiority and demonstrating superiority with continuous rhythm monitoring [108]. The SPHERE Per-AF trial randomized 420 patients with persistent AF to a dual-energy lattice-tip catheter capable of delivering pulsed field or RF energy versus conventional RF ablation, demonstrating non-inferior effectiveness with 1-year success rates of 74% and 66%, respectively [109]. More recently, the BEAT PAROX-AF randomized trial of 292 patients with paroxysmal AF showed comparable single-procedure success at 12 months between PFA and RF ablation, 77% vs. 78% [110]. Collectively, these trials demonstrate that catheter-based PFA achieves similar efficacy to established thermal ablation technologies while offering procedural efficiency and a favorable safety profile.

The MANIFEST-PF trial, which included 1700 patients, demonstrated acute PVI success rates above 99% with 78% of patients free from atrial arrhythmias at one year. Among those, patients with persistent and long-standing persistent AF, one-year success rates were 71% and 73%, respectively, while 45% of recurrences occurred in these more advanced subgroups [111]. This represents a clinically relevant limitation in catheter-based PFA, where acute conduction block does not necessarily predict long-term durability and may contribute to late reconnection and conduction gaps identified at repeat procedures. The larger MANIFEST-17K registry, encompassing over 17,000 patients, confirmed these results with similarly high acute success and durable rhythm control [112]. Importantly, both studies reported excellent safety outcomes, including the absence of atrioesophageal fistula and low incidence of phrenic nerve injury and stroke, complications that have historically limited thermal ablation.

Additional experience with catheter-based PFA has been reported in prospective multicenter studies. The inspIRE trial evaluated an integrated biphasic PFA system in 226 patients with drug-refractory paroxysmal AF, achieving acute PVI in 100% of patients and approximately 71% freedom from atrial arrhythmia at one year. Importantly, no primary adverse events were reported, and there were no cases of esophageal injury or pulmonary vein stenosis [113]. Similarly, the PULSED-AF trial enrolled 300 patients with paroxysmal and persistent AF treated with a circular PFA catheter system, demonstrating one-year freedom from AF of 66% in paroxysmal AF and 55% in persistent AF [114]. These studies further support the feasibility, efficacy, and favorable safety profile of catheter-based PFA across multiple device platforms and clinical settings, further support the feasibility, efficacy, and favorable safety profile of catheter-based PFA across multiple device platforms and clinical settings.

These findings underscore the continued challenge of reliably treating advanced atrial disease but also highlight the potential of PFA. With its rapid lesion formation and safety profile, PFA represents a promising non-thermal alternative that may expand the role of ablation therapy across both surgical and catheter-based platforms.

## 6. Future Directions

Advances in energy delivery continue to shape the evolution of surgical AF ablation. Bipolar RF and cryoablation are the most widely used modalities due to their versatility and proven long-term durability. Decades of clinical experience have demonstrated that both can achieve durable rhythm control when applied effectively, yet each carries intrinsic limitations. Successful lesion formation depends on consistent tissue contact and conductive heat transfer, making outcomes susceptible to variable wall thickness, epicardial fat, and the heat sink resulting from the circulating intracavitary blood volume.

PFA may overcome some of these challenges by introducing a non-thermal mechanism of tissue ablation. PFA uses short, high-voltage electrical pulses to create IRE. Catheter-based PFA has already achieved Conformité Européenne (European Conformity) Mark approval in Europe and recently received FDA approval in the United States for paroxysmal and persistent AF, accelerating clinical adoption [115,116,117]. Surgical applications of PFA remain in early stages, but initial studies are underway. In Europe, pilot feasibility trials are evaluating epicardial PFA systems in patients undergoing concomitant cardiac surgery, with endpoints including acute conduction block, operative efficiency, and safety outcomes [118]. Key questions remain regarding the long-term durability of PFA lesions, histologic validation, and overall cost-effectiveness. As larger clinical trials are conducted, comparative data will be essential to define how PFA integrates with or replaces existing energy sources in surgical practice.

Looking ahead, the landscape of surgical AF ablation is likely to become more multimodal. Thermal ablation will continue to provide reliable options with well-established outcomes, particularly for centers experienced in these techniques. PFA represents the next phase of innovation, with the potential to achieve transmural epicardial ablation on the beating heart, though its ultimate role will depend on clinical trial outcomes. The advancement of ablation technologies may expand the reach of surgical ablation, bringing durable rhythm control to a broader population of patients with AF.

## 7. Conclusions

Surgical ablation is the most effective and durable treatment for AF, particularly in persistent and long-standing persistent AF. The evolution from the original cut-and-sew CMP to modern energy-based lesion creation has simplified procedures and reduced morbidity while maintaining excellent rhythm outcomes. Bipolar RF and cryoablation form the foundation of contemporary surgical ablation. Minimally invasive and hybrid approaches have expanded access to durable rhythm control. They seek to replicate the CMP lesion set through smaller incisions, offering shorter recovery and lower morbidity. Hybrid ablation combines surgical ablation with catheter-based ablation to ensure complete left atrial lesion sets and improve rhythm durability compared to catheter ablation alone.

PFA represents the next step in surgical AF therapy. By inducing IRE using non-thermal energy, PFA creates rapid, tissue-selective lesions while minimizing collateral damage. Preclinical studies suggest excellent procedural efficacy. As PFA continues to develop, surgical ablation is likely to become more multimodal, combining established thermal techniques with PFA energy sources to expand safe and effective rhythm control to more patients.

## Abbreviations

AF, atrial fibrillation; RF, radiofrequency; PFA, pulsed-field ablation; LAA, left atrial appendage; LA, left atrium; IRE, irreversible electroporation; STS, Society of Thoracic Surgeons; FDA, Food and Drug Administration; CMP, Cox-Maze procedure; PVI, Pulmonary Vein Isolation.

## References

[1] Joglar JA, Chung MK, Armbruster AL, Benjamin EJ, Chyou JY, Cronin EM (2024). 2023 ACC/AHA/ACCP/HRS Guideline for the Diagnosis and Management of Atrial Fibrillation: A Report of the American College of Cardiology/American Heart Association Joint Committee on Clinical Practice Guidelines. Circulation.

[2] Tzeis S, Gerstenfeld EP, Kalman J, Saad EB, Sepehri Shamloo A, Andrade JG (2024). 2024 European Heart Rhythm Association/Heart Rhythm Society/Asia Pacific Heart Rhythm Society/Latin American Heart Rhythm Society expert consensus statement on catheter and surgical ablation of atrial fibrillation. Europace : European Pacing, Arrhythmias, and Cardiac Electrophysiology : Journal of the Working Groups on Cardiac Pacing, Arrhythmias, and Cardiac Cellular Electrophysiology of the European Society of Cardiology.

[3] Wyler von Ballmoos MC, Hui DS, Mehaffey JH, Malaisrie SC, Vardas PN, Gillinov AM (2024). The Society of Thoracic Surgeons 2023 Clinical Practice Guidelines for the Surgical Treatment of Atrial Fibrillation. The Annals of Thoracic Surgery.

[4] Rottner L, Metzner A (2023). Atrial Fibrillation Ablation: Current Practice and Future Perspectives. Journal of Clinical Medicine.

[5] Wazni OM, Dandamudi G, Sood N, Hoyt R, Tyler J, Durrani S (2021). Cryoballoon Ablation as Initial Therapy for Atrial Fibrillation. The New England Journal of Medicine.

[6] Sørensen SK, Johannessen A, Worck R, Hansen ML, Hansen J (2021). Radiofrequency Versus Cryoballoon Catheter Ablation for Paroxysmal Atrial Fibrillation: Durability of Pulmonary Vein Isolation and Effect on Atrial Fibrillation Burden: The RACE-AF Randomized Controlled Trial. Circulation. Arrhythmia and Electrophysiology.

[7] Campbell LA, Ammon JP, Kombathula R, Muhammad N, Jackson CD (2025). New atrial fibrillation guideline: Modify risk, control rhythm, prevent progression. Cleveland Clinic Journal of Medicine.

[8] Markman TM, Hyman MC, Kumareswaran R, Arkles JS, Santangeli P, Schaller RD (2020). Durability of posterior wall isolation after catheter ablation among patients with recurrent atrial fibrillation. Heart Rhythm.

[9] Al-Hijji MA, Deshmukh AJ, Yao X, Mwangi R, Sangaralingham LR, Friedman PA (2016). Trends and predictors of repeat catheter ablation for atrial fibrillation. American Heart Journal.

[10] Doll N, Weimar T, Kosior DA, Bulava A, Mokracek A, Mönnig G (2023). Efficacy and safety of hybrid epicardial and endocardial ablation versus endocardial ablation in patients with persistent and longstanding persistent atrial fibrillation: a randomised, controlled trial. EClinicalMedicine.

[11] Tilz RR, Rillig A, Thum AM, Arya A, Wohlmuth P, Metzner A (2012). Catheter ablation of long-standing persistent atrial fibrillation: 5-year outcomes of the Hamburg Sequential Ablation Strategy. Journal of the American College of Cardiology.

[12] Henn MC, Lancaster TS, Miller JR, Sinn LA, Schuessler RB, Moon MR (2015). Late outcomes after the Cox maze IV procedure for atrial fibrillation. The Journal of Thoracic and Cardiovascular Surgery.

[13] Weimar T, Schena S, Bailey MS, Maniar HS, Schuessler RB, Cox JL (2012). The cox-maze procedure for lone atrial fibrillation: a single-center experience over 2 decades. Circulation. Arrhythmia and Electrophysiology.

[14] McGilvray MMO, Bakir NH, Kelly MO, Perez SC, Sinn LA, Schuessler RB (2021). Efficacy of the stand-alone Cox-Maze IV procedure in patients with longstanding persistent atrial fibrillation. Journal of Cardiovascular Electrophysiology.

[15] Khiabani AJ, MacGregor RM, Bakir NH, Manghelli JL, Sinn LA, Maniar HS (2022). The long-term outcomes and durability of the Cox-Maze IV procedure for atrial fibrillation. The Journal of Thoracic and Cardiovascular Surgery.

[16] Cox JL, Schuessler RB, Boineau JP (2000). The development of the Maze procedure for the treatment of atrial fibrillation. Seminars in Thoracic and Cardiovascular Surgery.

[17] Gaynor SL, Diodato MD, Prasad SM, Ishii Y, Schuessler RB, Bailey MS (2004). A prospective, single-center clinical trial of a modified Cox maze procedure with bipolar radiofrequency ablation. The Journal of Thoracic and Cardiovascular Surgery.

[18] Philpott JM, Zemlin CW, Cox JL, Stirling M, Mack M, Hooker RL (2015). The ABLATE Trial: Safety and Efficacy of Cox Maze-IV Using a Bipolar Radiofrequency Ablation System. The Annals of Thoracic Surgery.

[19] Almousa A, Mehaffey JH, Wei LM, Simsa A, Hayanga JWA, Cook C (2023). Robotic-assisted cryothermic Cox maze for persistent atrial fibrillation: Longitudinal follow-up. The Journal of Thoracic and Cardiovascular Surgery.

[20] Lawrance CP, Henn MC, Miller JR, Sinn LA, Schuessler RB, Maniar HS (2014). A minimally invasive Cox maze IV procedure is as effective as sternotomy while decreasing major morbidity and hospital stay. The Journal of Thoracic and Cardiovascular Surgery.

[21] Ad N, Holmes SD, Friehling T (2017). Minimally Invasive Stand-Alone Cox Maze Procedure for Persistent and Long-Standing Persistent Atrial Fibrillation: Perioperative Safety and 5-Year Outcomes. Circulation. Arrhythmia and Electrophysiology.

[22] MOE GK, ABILDSKOV JA (1959). Atrial fibrillation as a self-sustaining arrhythmia independent of focal discharge. American Heart Journal.

[23] Williams JM, Ungerleider RM, Lofland GK, Cox JL, Sabiston DC (1980). Left atrial isolation: new technique for the treatment of supraventricular arrhythmias. The Journal of Thoracic and Cardiovascular Surgery.

[24] Cox JL, Schuessler RB, D'Agostino HJ, Stone CM, Chang BC, Cain ME (1991). The surgical treatment of atrial fibrillation. III. Development of a definitive surgical procedure. The Journal of Thoracic and Cardiovascular Surgery.

[25] Cox JL (1991). The surgical treatment of atrial fibrillation. IV. Surgical technique. The Journal of Thoracic and Cardiovascular Surgery.

[26] Cox JL, Schuessler RB, Lappas DG, Boineau JP (1996). An 8 1/2-year clinical experience with surgery for atrial fibrillation. Annals of Surgery.

[27] Cox JL, Canavan TE, Schuessler RB, Cain ME, Lindsay BD, Stone C (1991). The surgical treatment of atrial fibrillation. II. Intraoperative electrophysiologic mapping and description of the electrophysiologic basis of atrial flutter and atrial fibrillation. The Journal of Thoracic and Cardiovascular Surgery.

[28] Cox JL, Boineau JP, Schuessler RB, Kater KM, Lappas DG (1993). Five-year experience with the maze procedure for atrial fibrillation. The Annals of Thoracic Surgery.

[29] Prasad SM, Maniar HS, Camillo CJ, Schuessler RB, Boineau JP, Sundt TM (2003). The Cox maze III procedure for atrial fibrillation: long-term efficacy in patients undergoing lone versus concomitant procedures. The Journal of Thoracic and Cardiovascular Surgery.

[30] Schuessler RB, Lee AM, Melby SJ, Voeller RK, Gaynor SL, Sakamoto SI (2009). Animal studies of epicardial atrial ablation. Heart Rhythm.

[31] Ruaengsri C, Schill MR, Khiabani AJ, Schuessler RB, Melby SJ, Damiano RJ (2018). The Cox-maze IV procedure in its second decade: still the gold standard?. European Journal of Cardio-thoracic Surgery : Official Journal of the European Association for Cardio-thoracic Surgery.

[32] Ad N, Henry L, Friehling T, Wish M, Holmes SD (2013). Minimally invasive stand-alone Cox-maze procedure for patients with nonparoxysmal atrial fibrillation. The Annals of Thoracic Surgery.

[33] Robertson JO, Saint LL, Leidenfrost JE, Damiano RJ (2014). Illustrated techniques for performing the Cox-Maze IV procedure through a right mini-thoracotomy. Annals of Cardiothoracic Surgery.

[34] Roberts HG, Wei LM, Dhamija A, Cook CC, Badhwar V (2021). Robotic assisted cryothermic biatrial Cox-Maze. Journal of Cardiovascular Electrophysiology.

[35] Badhwar V (2023). Robotic-assisted biatrial Cox-maze ablation for atrial fibrillation. The Journal of Thoracic and Cardiovascular Surgery.

[36] DeLurgio DB, Crossen KJ, Gill J, Blauth C, Oza SR, Magnano AR (2020). Hybrid Convergent Procedure for the Treatment of Persistent and Long-Standing Persistent Atrial Fibrillation: Results of CONVERGE Clinical Trial. Circulation. Arrhythmia and Electrophysiology.

[37] van der Heijden CAJ, Weberndörfer V, Vroomen M, Luermans JG, Chaldoupi SM, Bidar E (2023). Hybrid Ablation Versus Repeated Catheter Ablation in Persistent Atrial Fibrillation: A Randomized Controlled Trial. JACC. Clinical Electrophysiology.

[38] Doll N, Weimar T, Kosior DA, Bulava A, Mokracek A, Mönnig G (2025). Durable effectiveness and safety of hybrid ablation versus catheter ablation: 2-year results from the randomized CEASE-AF trial†. European Journal of Cardio-thoracic Surgery : Official Journal of the European Association for Cardio-thoracic Surgery.

[39] Lawrance CP, Henn MC, Damiano RJ (2015). Surgical ablation for atrial fibrillation: techniques, indications, and results. Current Opinion in Cardiology.

[40] Melby SJ, Schuessler RB, Damiano RJ (2013). Ablation technology for the surgical treatment of atrial fibrillation. ASAIO Journal (American Society for Artificial Internal Organs : 1992).

[41] Klinkenberg TJ, Ahmed S, Ten Hagen A, Wiesfeld ACP, Tan ES, Zijlstra F (2009). Feasibility and outcome of epicardial pulmonary vein isolation for lone atrial fibrillation using minimal invasive surgery and high intensity focused ultrasound. Europace : European Pacing, Arrhythmias, and Cardiac Electrophysiology : Journal of the Working Groups on Cardiac Pacing, Arrhythmias, and Cardiac Cellular Electrophysiology of the European Society of Cardiology.

[42] Lall SC, Damiano RJ (2007). Surgical ablation devices for atrial fibrillation. Journal of Interventional Cardiac Electrophysiology : an International Journal of Arrhythmias and Pacing.

[43] Thomas SP, Guy DJR, Boyd AC, Eipper VE, Ross DL, Chard RB (2003). Comparison of epicardial and endocardial linear ablation using handheld probes. The Annals of Thoracic Surgery.

[44] Yates TA, McGilvray M, Schill MR, Barron L, Razo N, Roberts HG (2023). Performance of an Irrigated Bipolar Radiofrequency Ablation Clamp on Explanted Human Hearts. The Annals of Thoracic Surgery.

[45] Yates TA, McGilvray M, Razo N, McElligott S, Melby SJ, Zemlin C (2022). Efficacy of a Novel Bipolar Radiofrequency Clamp: An Acute Porcine Model. Innovations (Philadelphia, Pa.).

[46] Khiabani AJ, MacGregor RM, Manghelli JL, Ruaengsri C, Carter DI, Melby SJ (2020). Bipolar Radiofrequency Ablation on Explanted Human Hearts: How to Ensure Transmural Lesions. The Annals of Thoracic Surgery.

[47] Lee AM, Aziz A, Clark KL, Schuessler RB, Damiano RJ (2012). Chronic performance of a novel radiofrequency ablation device on the beating heart: Limitations of conduction delay to assess transmurality. The Journal of Thoracic and Cardiovascular Surgery.

[48] Khalpey Z, Aslam U, Kumar U, Epting L (2024). First-in-Man Use of Intraoperative Electrophysiological Mapping to Evaluate the Efficacy of the EnCompass Clamp During a Cox-IV Maze Procedure. Cureus.

[49] McGilvray MMO, Barron L, Yates TAE, Zemlin CW, Damiano RJ (2022). The Cox-Maze procedure: What lesions and why. JTCVS Techniques.

[50] Moon MR, Kachroo P (2024). How we do it: Biatrial maze. JTCVS Structural and Endovascular.

[51] Arya A, Di Biase L, Bazán V, Berruezo A, d'Avila A, Della Bella P (2025). Epicardial ventricular arrhythmia ablation: a clinical consensus statement of the European Heart Rhythm Association of the European Society of Cardiology and the Heart Rhythm Society, the Asian Pacific Heart Rhythm Society, the Latin American Heart Rhythm Society, and the Canadian Heart Rhythm Society. Europace : European Pacing, Arrhythmias, and Cardiac Electrophysiology : Journal of the Working Groups on Cardiac Pacing, Arrhythmias, and Cardiac Cellular Electrophysiology of the European Society of Cardiology.

[52] TAYLOR CB, DAVIS CB, VAWTER GF, HASS GM (1951). Controlled myocardial injury produced by a hypothermal method. Circulation.

[53] Gammie JS, Laschinger JC, Brown JM, Poston RS, Pierson RN, Romar LG (2005). A multi-institutional experience with the CryoMaze procedure. The Annals of Thoracic Surgery.

[54] McCarthy PM, Cox JL, Kruse J, Elenbaas C, Andrei AC (2024). One hundred percent utilization of a modified CryoMaze III procedure for atrial fibrillation with mitral surgery. The Journal of Thoracic and Cardiovascular Surgery.

[55] Snyder KK, Baust JG, Baust JM, Gage AA, Bredikis AJ, Wilber DJ (2011). Mechanisms of Cryoablation. Cryoablation of Cardiac Arrhythmias.

[56] Steinbach JP, Weissenberger J, Aguzzi A (1999). Distinct phases of cryogenic tissue damage in the cerebral cortex of wild-type and c-fos deficient mice. Neuropathology and Applied Neurobiology.

[57] Cox JL, Malaisrie SC, Churyla A, Mehta C, Kruse J, Kislitsina ON (2021). Cryosurgery for Atrial Fibrillation: Physiologic Basis for Creating Optimal Cryolesions. The Annals of Thoracic Surgery.

[58] Schill MR, Melby SJ, Speltz M, Breitbach M, Schuessler RB, Damiano RJ (2017). Evaluation of a Novel Cryoprobe for Atrial Ablation in a Chronic Ovine Model. The Annals of Thoracic Surgery.

[59] Ad N, Damiano RJ, Badhwar V, Calkins H, La Meir M, Nitta T (2017). Expert consensus guidelines: Examining surgical ablation for atrial fibrillation. The Journal of Thoracic and Cardiovascular Surgery.

[60] Lustgarten DL, Bell S, Hardin N, Calame J, Spector PS (2005). Safety and efficacy of epicardial cryoablation in a canine model. Heart Rhythm.

[61] Tabaja C, Younis A, Hussein AA, Taigen TL, Nakagawa H, Saliba WI (2023). Catheter-Based Electroporation: A Novel Technique for Catheter Ablation of Cardiac Arrhythmias. JACC. Clinical Electrophysiology.

[62] Davalos RV, Mir ILM, Rubinsky B (2005). Tissue ablation with irreversible electroporation. Annals of Biomedical Engineering.

[63] Batista Napotnik T, Polajžer T, Miklavčič D (2021). Cell death due to electroporation - A review. Bioelectrochemistry (Amsterdam, Netherlands).

[64] Rubinsky L, Guenther E, Mikus P, Stehling M, Rubinsky B (2016). Electrolytic Effects During Tissue Ablation by Electroporation. Technology in Cancer Research & Treatment.

[65] Wittkampf FH, van Driel VJ, van Wessel H, Vink A, Hof IE, Gründeman PF (2011). Feasibility of electroporation for the creation of pulmonary vein ostial lesions. Journal of Cardiovascular Electrophysiology.

[66] Reddy VY, Neuzil P, Koruth JS, Petru J, Funosako M, Cochet H (2019). Pulsed Field Ablation for Pulmonary Vein Isolation in Atrial Fibrillation. Journal of the American College of Cardiology.

[67] Reddy VY, Dukkipati SR, Neuzil P, Anic A, Petru J, Funasako M (2021). Pulsed Field Ablation of Paroxysmal Atrial Fibrillation: 1-Year Outcomes of IMPULSE, PEFCAT, and PEFCAT II. JACC. Clinical Electrophysiology.

[68] Jeong S, Kim H, Park J, Kim KW, Sim SB, Chung JH (2021). Evaluation of electroporated area using 2,3,5-triphenyltetrazolium chloride in a potato model. Scientific Reports.

[69] Sullivan AP, Aguilar M, Laksman Z (2025). Pulsed Field Ablation: A Review of Preclinical and Clinical Studies. Bioengineering (Basel, Switzerland).

[70] Wittkampf FHM, van Es R, Neven K (2018). Electroporation and its Relevance for Cardiac Catheter Ablation. JACC. Clinical Electrophysiology.

[71] Hunter DW, Kostecki G, Fish JM, Jensen JA, Tandri H (2021). In Vitro Cell Selectivity of Reversible and Irreversible: Electroporation in Cardiac Tissue. Circulation. Arrhythmia and Electrophysiology.

[72] Baena-Montes JM, O'Halloran T, Clarke C, Donaghey K, Dunne E, O'Halloran M (2022). Electroporation Parameters for Human Cardiomyocyte Ablation In Vitro. Journal of Cardiovascular Development and Disease.

[73] Kaminska I, Kotulska M, Stecka A, Saczko J, Drag-Zalesinska M, Wysocka T (2012). Electroporation-induced changes in normal immature rat myoblasts (H9C2). General Physiology and Biophysics.

[74] Howard B, Haines DE, Verma A, Packer D, Kirchhof N, Barka N (2020). Reduction in Pulmonary Vein Stenosis and Collateral Damage With Pulsed Field Ablation Compared With Radiofrequency Ablation in a Canine Model. Circulation. Arrhythmia and Electrophysiology.

[75] Reddy VY, Koruth J, Jais P, Petru J, Timko F, Skalsky I (2018). Ablation of Atrial Fibrillation With Pulsed Electric Fields: An Ultra-Rapid, Tissue-Selective Modality for Cardiac Ablation. JACC. Clinical Electrophysiology.

[76] Reddy VY, Petru J, Funasako M, Kopriva K, Hala P, Chovanec M (2022). Coronary Arterial Spasm During Pulsed Field Ablation to Treat Atrial Fibrillation. Circulation.

[77] Yi J, Yu J, Procasky S, Obiarinze R, Rahimi M, Arif B (2026). Nanosecond pulsed field ablation: Feasibility of creating the Cox-maze lesion set on the beating heart. The Journal of Thoracic and Cardiovascular Surgery.

[78] van Driel VJHM, Neven K, van Wessel H, Vink A, Doevendans PAFM, Wittkampf FHM (2015). Low vulnerability of the right phrenic nerve to electroporation ablation. Heart Rhythm.

[79] Neven K, van Es R, van Driel V, van Wessel H, Fidder H, Vink A (2017). Acute and Long-Term Effects of Full-Power Electroporation Ablation Directly on the Porcine Esophagus. Circulation. Arrhythmia and Electrophysiology.

[80] Koruth JS, Kuroki K, Kawamura I, Brose R, Viswanathan R, Buck ED (2020). Pulsed Field Ablation Versus Radiofrequency Ablation: Esophageal Injury in a Novel Porcine Model. Circulation. Arrhythmia and Electrophysiology.

[81] Seiler J, Roberts-Thomson KC, Raymond JM, Vest J, Delacretaz E, Stevenson WG (2008). Steam pops during irrigated radiofrequency ablation: feasibility of impedance monitoring for prevention. Heart Rhythm.

[82] van Es R, Groen MHA, Stehouwer M, Doevendans PA, Wittkampf FHM, Neven K (2019). In vitro analysis of the origin and characteristics of gaseous microemboli during catheter electroporation ablation. Journal of Cardiovascular Electrophysiology.

[83] Nies M, Koruth JS, Mlček M, Watanabe K, Tibenská VC, Královec Š (2024). Hemolysis After Pulsed Field Ablation: Impact of Lesion Number and Catheter-Tissue Contact. Circulation. Arrhythmia and Electrophysiology.

[84] Gianni C, Al-Ahmad A, Elchouemi M, La Fazia VM, Mohanty S, Allison JD (2025). Pulsed Field Ablation-Related Hemolysis: Comparison Between Technologies. Circulation. Arrhythmia and Electrophysiology.

[85] Martinez J, Challapalli M, Hutchinson M, Ibrahim M, Shen C, Klewer J (2026). Renal safety of high-dose pulsed field ablation of atrial fibrillation: A prospective real-world analysis. Heart Rhythm.

[86] Tamirisa KP, Sanchez JE, La Fazia VM, Gianni C, Serpa F, Mohanty S (2025). Pulsed field ablation-related hemoglobinuria and acute kidney injury: Insights and strategies for effective management. American Heart Journal.

[87] Boersma LV, van der Voort P, Debruyne P, Dekker L, Simmers T, Rossenbacker T (2016). Multielectrode Pulmonary Vein Isolation Versus Single Tip Wide Area Catheter Ablation for Paroxysmal Atrial Fibrillation: A Multinational Multicenter Randomized Clinical Trial. Circulation. Arrhythmia and Electrophysiology.

[88] Wood MA, Fuller IA (2002). Acute and chronic electrophysiologic changes surrounding radiofrequency lesions. Journal of Cardiovascular Electrophysiology.

[89] Lapenna E, De Bonis M, Giambuzzi I, Del Forno B, Ruggeri S, Cireddu M (2020). Long-term Outcomes of Stand-Alone Maze IV for Persistent or Long-standing Persistent Atrial Fibrillation. The Annals of Thoracic Surgery.

[90] MacGregor RM, Bakir NH, Pedamallu H, Sinn LA, Maniar HS, Melby SJ (2022). Late results after stand-alone surgical ablation for atrial fibrillation. The Journal of Thoracic and Cardiovascular Surgery.

[91] Calkins H, Hindricks G, Cappato R, Kim YH, Saad EB, Aguinaga L (2017). 2017 HRS/EHRA/ECAS/APHRS/SOLAECE expert consensus statement on catheter and surgical ablation of atrial fibrillation. Heart Rhythm.

[92] McClure GR, Belley-Cote EP, Jaffer IH, Dvirnik N, An KR, Fortin G (2018). Surgical ablation of atrial fibrillation: a systematic review and meta-analysis of randomized controlled trials. Europace : European Pacing, Arrhythmias, and Cardiac Electrophysiology : Journal of the Working Groups on Cardiac Pacing, Arrhythmias, and Cardiac Cellular Electrophysiology of the European Society of Cardiology.

[93] Montané B, Zhang S, Wolfe JD, Prime S, Luo C, Cooper DH (2025). Catheter and Surgical Ablation for Atrial Fibrillation : A Systematic Review and Meta-analysis. Annals of Internal Medicine.

[94] Osmancik P, Budera P, Talavera D, Hlavicka J, Herman D, Holy J (2019). Five-year outcomes in cardiac surgery patients with atrial fibrillation undergoing concomitant surgical ablation versus no ablation. The long-term follow-up of the PRAGUE-12 Study. Heart Rhythm.

[95] Musharbash FN, Schill MR, Sinn LA, Schuessler RB, Maniar HS, Moon MR (2018). Performance of the Cox-maze IV procedure is associated with improved long-term survival in patients with atrial fibrillation undergoing cardiac surgery. The Journal of Thoracic and Cardiovascular Surgery.

[96] Mitrovic I, Eszlari E, Cvorak A, Liebold A, Rastan A, Grubitzsch H (2024). Epicardial and endocardial surgical ablation of atrial fibrillation: outcomes from CASE-AF Registry. Interdisciplinary Cardiovascular and Thoracic Surgery.

[97] Ju MH, Huh JH, Lee CH, Kim HJ, Je HG, Kim JB (2019). Robotic-Assisted Surgical Ablation of Atrial Fibrillation Combined With Mitral Valve Surgery. The Annals of Thoracic Surgery.

[98] Boersma LVA, Castella M, van Boven W, Berruezo A, Yilmaz A, Nadal M (2012). Atrial fibrillation catheter ablation versus surgical ablation treatment (FAST): a 2-center randomized clinical trial. Circulation.

[99] Adiyaman A, Buist TJ, Beukema RJ, Smit JJJ, Delnoy PPHM, Hemels MEW (2018). Randomized Controlled Trial of Surgical Versus Catheter Ablation for Paroxysmal and Early Persistent Atrial Fibrillation. Circulation. Arrhythmia and Electrophysiology.

[100] Saini A, Hu YL, Kasirajan V, Han FT, Khan MZ, Wolfe L (2017). Long-term outcomes of minimally invasive surgical ablation for atrial fibrillation: A single-center experience. Heart Rhythm.

[101] Bisleri G, Pandey AK, Verma S, Ali Hassan SM, Yanagawa B, Khandaker M (2023). Combined Minimally Invasive Surgical and Percutaneous Catheter Ablation of Atrial Fibrillation: JACC Review Topic of the Week. Journal of the American College of Cardiology.

[102] Van Der Heijden CAJ, Vroomen M, Weberndorfer CV, Luermans JGLM, Chaldoupi SM, Bidar E (2024). Hybrid ablation versus repeated catheter ablation in persistent atrial fibrillation: long-term results of the HARTCAP-AF trial. Europace.

[103] Serra F, Philpott JM, Neuber JU, Shih E, Etheridge JC, Varghese F (2023). Nanosecond Pulsed Electric Field Ablation With a Bipolar Clamp Creates Durable Transmural Lesions in Cardiac Tissue. Circulation. Arrhythmia and Electrophysiology.

[104] Varghese F, Philpott JM, Neuber JU, Hargrave B, Zemlin CW (2023). Surgical Ablation of Cardiac Tissue with Nanosecond Pulsed Electric Fields in Swine. Cardiovascular Engineering and Technology.

[105] Yu J, Yi J, Nikolaisen G, Wilson LD, Schill MR, Damiano RJ (2025). Efficacy of a surgical cardiac ablation clamp using nanosecond pulsed electric fields: An acute porcine model. The Journal of Thoracic and Cardiovascular Surgery.

[106] Dunnington GH, Waterford SD, Uecker D, Johnston L, Danitz D, Crompton A (2025). The performance of a new nanosecond pulsed-field ablation surgical clamp in the ablation of cardiac tissue: A chronic porcine model. The Journal of Thoracic and Cardiovascular Surgery.

[107] Reddy VY, Gerstenfeld EP, Natale A, Whang W, Cuoco FA, Patel C (2023). Pulsed Field or Conventional Thermal Ablation for Paroxysmal Atrial Fibrillation. The New England Journal of Medicine.

[108] Reichlin T, Kueffer T, Badertscher P, Jüni P, Knecht S, Thalmann G (2025). Pulsed Field or Cryoballoon Ablation for Paroxysmal Atrial Fibrillation. The New England Journal of Medicine.

[109] Anter E, Mansour M, Nair DG, Sharma D, Taigen TL, Neuzil P (2024). Dual-energy lattice-tip ablation system for persistent atrial fibrillation: a randomized trial. Nature Medicine.

[110] Jais P, Neuzil P, Scherr D, Frison E, Knecht S, Boveda S (2026). Pulsed field vs radiofrequency ablation for paroxysmal atrial fibrillation: the BEAT PAROX-AF trial. European Heart Journal.

[111] Turagam MK, Neuzil P, Schmidt B, Reichlin T, Neven K, Metzner A (2023). Safety and Effectiveness of Pulsed Field Ablation to Treat Atrial Fibrillation: One-Year Outcomes From the MANIFEST-PF Registry. Circulation.

[112] Ekanem E, Neuzil P, Reichlin T, Kautzner J, van der Voort P, Jais P (2024). Safety of pulsed field ablation in more than 17,000 patients with atrial fibrillation in the MANIFEST-17K study. Nature Medicine.

[113] Duytschaever M, De Potter T, Grimaldi M, Anic A, Vijgen J, Neuzil P (2023). Paroxysmal Atrial Fibrillation Ablation Using a Novel Variable-Loop Biphasic Pulsed Field Ablation Catheter Integrated With a 3-Dimensional Mapping System: 1-Year Outcomes of the Multicenter inspIRE Study. Circulation. Arrhythmia and Electrophysiology.

[114] Verma A, Haines DE, Boersma LV, Sood N, Natale A, Marchlinski FE (2023). Pulsed Field Ablation for the Treatment of Atrial Fibrillation: PULSED AF Pivotal Trial. Circulation.

[115] Kyriagis A (2023). Medtronic creates history with FDA approval of its novel PulseSelect™ Pulsed Field Ablation System to treat atrial fibrillation. https://news.medtronic.com/2023-12-13-Medtronic-creates-history-with-FDA-approval-of-its-novel-PulseSelect-TM-Pulsed-Field-Ablation-System-to-treat-atrial-fibrillation.

[116] Heath C, HEEN A (2025). Abbott Receives CE Mark for its Volt™ Pulsed Field Ablation System to Treat Patients with Abnormal Heart Rhythms. https://abbott.mediaroom.com/2025-03-27-Abbott-Receives-CE-Mark-for-its-Volt-TM-Pulsed-Field-Ablation-System-to-Treat-Patients-with-Abnormal-Heart-Rhythms.

[117] Bailey (2024). Boston Scientific receives FDA approval for FARAPULSE™ Pulsed Field Ablation System. https://news.bostonscientific.com/2024-01-31-Boston-Scientific-Receives-FDA-Approval-for-FARAPULSE-TM-Pulsed-Field-Ablation-System.

[118] Musikantow DR, Reddy VY, Shaburishvili T, van Zyl M, O'Brien B, Coffey K (2024). Epicardial Pulsed Field Ablation for the Treatment of Paroxysmal Atrial Fibrillation During Cardiac Surgery. JACC. Clinical Electrophysiology.

